# Understanding and assessing personality across cultures: A scoping review

**DOI:** 10.1371/journal.pone.0338521

**Published:** 2026-01-02

**Authors:** Hannah Sheppard, Boris Bizumic, Bruce Christensen, Conal Monaghan

**Affiliations:** School of Medicine and Psychology, Australian National University, Canberra, Australia; University of Glasgow, UNITED KINGDOM OF GREAT BRITAIN AND NORTHERN IRELAND

## Abstract

Previous reviews of personality trait instruments have questioned whether meaningful cross-cultural comparisons of personality can be made. Personality, however, extends beyond personality traits. The current scoping review utilized McAdams’s Three-Layer framework of personality to assess the cross-cultural validity of measures of traits, characteristic adaptations, and life narratives to deepen the understanding of whether personality can be effectively assessed across different cultures. 233 publications were identified from searching scientific databases, leading international journals in personality and assessment, and databases for specific personality instruments between the 23^rd^ of January and the 5^th^ of June 2024. The review identified models of personality that have been empirically or theoretically supported across different cultures, focusing on measures that are structurally equivalent across cultures and have strong validity estimates within non-Western cultures. This review principally focused on cross-cultural research published in English within the last 20 years (2004–2024), and concentrated on broad integrative models of personality, which investigate a myriad of personality constructs, such as personality traits, values, and beliefs. The majority of publications (59%) utilized personality trait models when assessing personality across-cultures. Although no instruments demonstrated evidence of full cross-cultural scalar invariance, the NEO-Personality Inventory Revised, and the International Personality Item Pool 120-item representation of the NEO-PI-R demonstrated the strongest evidence for validity and reliability in their cross-cultural personality trait assessment. The Schwartz values measure, the 21-item Portrait Values Questionnaire, also demonstrated acceptable psychometric properties and partial scalar measurement invariance across cultures. Some measures were found to perform well within specific cultures. Nonetheless, this review cautions against making inferences about differences in average levels of personality between cultures due to the lack of scalar invariance across nearly all personality measures.

## Introduction

Personality is a complex construct that situates an individual’s dispositional traits, characteristic adaptations (e.g., beliefs, values), and life narratives within their social and cultural context [1]. It has been found to predict various individual, interpersonal and institutional outcomes, including happiness, community involvement, criminal activity, and political ideology [2], as well as mortality, occupational outcomes, and academic performance [3,4]. As such, research into how personality influences individuals and groups is a central focus of psychology.

Given that culture describes shared history, knowledge, and social norms that contextualize personality, they are interdependent constructs and the assessment of personality requires an understanding of the relationships between the two [5]. However, previous reviews have reported limited evidence for the cross-cultural measurement invariance of instruments used to assess personality and questioned whether meaningful cross-cultural comparisons can be made (e.g., [6,7]). To contribute further to the understanding of assessing personality across cultures, this review sought to compare the psychometric validity of etic and emic personality trait measures, as well as instruments used to assess personality beyond traits (e.g., characteristic adaptations and life narratives). This review aimed to provide insight into which models of personality are useful for cross-cultural personality assessment and presents evidence for specific measures used in personality assessment within cultures.

Accurate assessment and comparison of personality across cultures requires the use of psychometrically sound instruments [8]. The instrument’s reliability is demonstrated through replicable or consistent instrument scores across time, raters, items, and/or different versions of the instrument. Its construct validity refers to the instrument’s capacity to accurately measure the construct it is intending to measure [9]. Measuring the validity of psychological scales has been a long-standing challenge. Evidence for validity can be ascertained by examining whether the scale measures the full breadth of each construct without measuring unnecessary content (content validity), demonstrates a pattern of associations consistent with the construct definition (convergent and divergent validity) and theoretically related variables (criterion validity), and predicts outcomes above and beyond that of other measures of the same construct (incremental validity). Both validity and reliability need to be considered when assessing the psychometric integrity of any instrument.

Moreover, to meaningfully compare personality across cultures, the instrument must demonstrate cross-cultural measurement invariance [10]. This means that the instrument’s structure is comparable across different cultural groups. For example, if agreeableness is being measured, the latent variables identified as components of agreeableness and scale items loading on each latent variable should be highly similar; otherwise, any differences could simply reflect cultural differences in interpreting these items rather than actual personality differences. Cultural norms can influence how personality traits manifest and are interpreted. For example, items that infer agreeableness (e.g., “Is helpful and not selfish with others”; Big Five Inventory; [11]) may be influenced by cultural values like renqing (a set of social norms which includes the requirement of generosity and charity towards others [12,13]) rather than the personality trait itself, resulting in a skewed distribution of scores for that group. Inclusion of items that are influenced by external variables in one group but not the other are likely to lead to inaccurate and non-replicable data regarding personality differences across cultures [6]. Measurement invariance ensures that comparisons, such as group averages or correlations with other variables, are valid and reflect true differences rather than measurement biases.

There are four levels of measurement invariance commonly tested (configural, metric, scalar, and residual; see S1 Table for the glossary), each of which allow for stronger level of inference regarding equivalence [14]. Establishing configural invariance is a prerequisite for moving on to more stringent tests. Configural invariance estimates the consistency of the overall factor structure between groups. That is, configural invariance assesses whether items group together similarly as factors (or traits) across groups. Next, metric invariance tests whether the strength of the relationship between specific items and the underlying factor/trait (i.e., factor loadings) is equivalent across groups. Scalar invariance goes a step further by testing whether differences in the average factor scores between groups reflect true differences in the underlying trait, rather than being due to systematic biases in how individuals from different cultures respond to the items. For example, by constraining the item intercepts (the baseline levels of responses) to be equivalent across groups, scalar invariance ensures that observed differences in Agreeableness are not confounded by different response tendencies, such as one culture consistently giving higher or lower scores regardless of the actual trait level. Finally, residual invariance examines whether the remaining unexplained variance, including specific variance (variance unique to each item not explained by the factor) and measurement error, are comparable across groups. Although not always included in measurement invariance analyses, residual invariance ensures that the precision and reliability of the measurement are similar across cultures, further reducing potential biases in interpreting group differences.

The method used to adapt an instrument for use in another culture or language may impact its cross-cultural validity. Instrument adaptation should involve consideration of the linguistic and cultural differences of the target population [15], and may require items, instructions, or response formats (e.g., the number and labels of Likert scale responses) to be translated, modified, or even replaced to ensure the intent of the original instrument is reflected in the adapted instrument [16]. The extent to which a personality instrument is adapted can vary substantially [17]. Church [17] specifies nine levels of test adaptation, which move from “imposed-etic” (no or literal translation of the instrument), through “indigenization from without” (adaptation of items or development of items that are more culturally relevant), to “indigenization from within (emic)” (the identification and development of indigenous constructs, response formats, and/or item content; p. 984). Although, each level of adaptation has its utility [15], literal translations, without consideration of cultural or contextual factors, can misconstrue the intent of the original instrument [16,18]. Fischer and colleagues [18] provide the example of the Extraversion indicator ‘talkative’, which can take on additional, negative meaning when translated into Portuguese, Samoan, or German. Without adaptation of the item, such as adding situational anchors or context, the trait indicator can lose equivalence across languages. Guidelines for the translation and adaptation of instruments, such as the International Test Commission’s Guidelines for Translating and Adapting Tests [15], should be followed to ensure appropriate and consistent adaption of personality instruments (for a review of the existing guidelines and recommendations, see [16]).

## Theoretical framework and review structure

Given the numerous conceptualizations of both personality and culture, we provide operational definitions of these constructs for the purposes of this review.

### Defining personality

Personality can be conceptualized as the relatively stable pattern of behaviors, thoughts, and emotions that characterize an individual [19,20]. Although usually approached from multiple perspectives (e.g., trait, psychodynamic, evolutionary, and humanistic), integrative frameworks [1,21,22] provide a more comprehensive understanding of these individual patterns, particularly in a cross-cultural context [23]. Given the focus of this review, we have largely focused on nomothetic (group focused) over ideographic models (individual focused) to describe personality [21].

McAdams’s three-layer framework of personality [24–26] is a strong example of an integrated approach, providing a general structure from multiple perspectives, permitting “both within… and between-person comparison[s]” (p. 207; [27]). McAdams’s framework has been substantiated by a large body of research to validate (e.g., [28–31]) and extend the original model [21,32]. The framework organizes older theories and contemporary personality theory and research under one rubric, making it a unifying and coherent framework that brings together various models into a layered structure. This allows for the approach to individual differences recommended by Roberts et al. [3], where a broad range of constructs beyond just personality traits can be considered.

McAdams’s framework incorporates other existing integrative frameworks, such as Mischel’s cognitive-affective personality system (CAPS; [33]) and McCrae and Costa’s [34] five-factor theory (FFT). The CAPS model focuses on the influence of the situation on behavior and proposes the “if… then…” behavioral signatures of personality [35]. Within this model, cognitive-affective units (CAUs; e.g., goals, beliefs, affects, competencies) are activated or inhibited as individuals move across situations, accounting for individual differences. Although a promising theoretical integration of personality, the CAPS lacks solid empirical support [27] and widespread acceptance by personality researchers, especially those who are concerned with personality assessment. Moreover, the CAUs and “if… then…” signatures are well represented within Layer 2 of McAdams’s model (discussed below). Similarly, the FFT, inspired by the five-factor model (FFM) of personality [36], suggests that personality centers around basic tendencies (traits, innate abilities, predispositions) and characteristic adaptations. Both of which are also subsumed in McAdams’s three-layer framework.

### McAdams’s three-layer framework

McAdams argues that personality can be defined via three developmental layers: (1) dispositional traits; (2) characteristic adaptations; and (3) life stories/narratives [24,26]. These layers emerge and evolve over time, providing an account for a person’s general tendencies, how they react and adapt to their social environment, and what their beliefs are about the meaning and purpose of their life [37].

**Layer 1.** Layer 1 consists of dispositional traits, which account for broad individual differences in behaviors, thoughts, and feelings across situations and time [26]. They provide a (typically) stable characterization of an individual’s personality and align with other trait models, such as the FFM [36] and HEXACO [38], including the maladaptive variants of traits that characterize personality disorders [39,40]. This layer encapsulates the trait approach’s strength in describing individual differences [21], including its strong empirical evidence, hierarchical structure, rank-order stability, and cross-cultural replicability [32,38,41–44]. Additionally, traits have biological foundations, with studies documenting their moderate heritability [30,45,46], including support for genetic influences independent of culture [47,48].

The dispositional traits of Layer 1 incorporate the FFM/FFT traits (i.e., Extraversion, Agreeableness, Conscientiousness, Neuroticism, and Openness; [36]), but this layer also recognizes that personality traits expand beyond the Big Five [49] and incorporates other plausible trait and temperament taxonomies. This includes the HEXACO model, which encompasses six basic traits: Honesty-Humility, Emotionality, Extraversion, Agreeableness (versus anger), Conscientiousness, and Openness to Experience. A related model is the regulatory model of temperament [50,51], which includes temperamentally caused temporal (briskness, perseveration, and rhythmicity) and energetic (sensory sensitivity, endurance, emotional reactivity, and activity) characteristics of behavior.

A persistent challenge in personality psychology is the assumption that concepts, theories, and methods developed in Western countries can be easily exported to non-Western cultural groups [52–54]. Western scales can easily neglect relevant psychological phenomena that are pronounced in other cultures. For example, the Big Five trait organization, particularly in relation to the Openness domain, has not always been successfully replicated outside Western samples [55,56]. As such, scales such as the Chinese Personality Assessment Inventory (CPAI) have been developed through an emic-etic approach to produce an inventory relevant to the target population while still maintaining relevant personality constructs found in etic (universal) scales [57].

The CPAI model proposes a four-factor structure comprising Social Potency, Dependability, Accommodation, and Interpersonal Relatedness [55,56,58]. Social potency reflects the extraversion and openness traits, measuring the sociability, leadership, innovation, value of change and diversity, and self-development aspects of personality [56,59]. Dependability measures traits of reliability, responsibility, and emotional stability, similar to those found in neuroticism and conscientiousness. Accommodation reflects agreeableness type traits, including acceptance of self and others and adaptation to the collective group. Interpersonal relatedness contains culturally specific elements of personality that reflect how Chinese people act in important relationships [60]. These include saving face (for self and others), avoiding face-to-face conflict, maintaining superficial harmony, and emphasizing reciprocity in relationships. This factor may represent a relationship-focused aspect of personality that is relevant in collectivist cultures [61].

Similarly, the Big Five measures have also been found to have lower levels of internal consistency and structural equivalence in African nations compared to other world regions [62–64]. Research found that Black South Africans used more specific behaviors and perceptions (e.g., “believes in the importance of helping”) and contextual information (e.g., “takes care of friends when they are unwell”) and fewer traits (e.g., “outgoing”) than White South Africans [65] when describing personality. For cultural groups where contextualization is particularly important when describing personality, the cultural influences on the conceptualization of dispositional traits likely limit the transferability of a Western-derived instrument. To address such issues, the South African Personality Inventory (SAPI) model was developed using an emic-etic approach through an investigation of personality-language used in the 11 official languages of South Africa [66].

Similar to the CPAI, the SAPI was developed to include culturally relevant traits alongside the existing Big Five traits. Research has demonstrated a six-factor structure containing Conscientiousness, Extraversion, Neuroticism, Openness, Negative Social-Relational Disposition, and Positive Social-Relational Disposition as well as equivalence across four major South African ethnic groups, White, African, Colored, and Indian [67]. The SAPI model does not include an agreeableness domain, which has been subsumed by the social-relational disposition factors [66]. The included universal traits conscientiousness, extraversion, neuroticism, and openness are largely the same as the conceptualizations in the FFM, despite minor conceptual variations. Negative Social-Relational Disposition reflects a contentious approach to social situations, such as being confrontational and rude, commonly criticizing or finding fault with others, and behaving as though they are better than other people [68]. The Positive Social-Relational Disposition trait reflects a positive approach to social relationships, for example, striving for harmonious resolutions in conflict, endeavoring not to harm others, being considerate of others needs and feelings, and engaging in the guiding, helping, and caring for of others.

**Layer 2.** Layer 2 consists of characteristic adaptations, which comprise the motivational (e.g., motives, projects, and goals), social-cognitive (e.g., values, beliefs, and cognitive schemas), and developmental (e.g., psychosocial stages and defense mechanisms) aspects of personality [1,26,29]. Characteristic adaptations describe the processes individuals employ to get what they want in life and are generally contextualized by time, place, situation, and/or social role. This layer involves a motivated agent who defines themselves by personal values, beliefs, ideologies, and goals, developed through individual agency and societal constraints [25]. This approach draws parallels to how characteristic adaptations are conceptualized in the FFT. Nevertheless, McAdams goes further by suggesting that although characteristic adaptations are related to personality traits, they should be seen as distinct from them. This distinction emphasizes the need for empirical evidence to support the connections between traits and adaptations, rather than assuming these relationships [24]. Whereas FFT describes the underlying tendency for how someone will behave and think across situations, Layer 2 describes how that person has adapted to their environment based on their experiences.

Given the breadth of characteristic adaptations, we have chosen to focus this review on values and beliefs because they have been consistently studied across cultures. Beliefs, defined as a mental state where something is accepted, trusted, and considered true without proof [69], vary in specificity and are characterized by the individuals and situations involved [70]. As such, many beliefs are narrow and only relevant to particular people and contexts. Personal values are widely described as broad, mostly stable life goals that are given importance, transcend situations, and provide guiding principles for a person or group [71,72]. Under this definition, the importance of a given value varies from person to person, with different people considering some values more important than others.

**Layer 3.** Layer 3 is the least researched aspect of the framework. It comprises the evolving personal narratives (life stories) that people construct to achieve a sense of meaning, identity, purpose, and coherence in their lives [23,26]. People’s self-narrative(s) reflect their entire lives, shaped by time and culture, as well as how their current identity is distinct from their past and imagined future. McAdams proposed that culture and personality are most closely tied in the life stories layer, where scripts derived from cultural history and contemporary popular culture inform the narratives people construct to make sense of their lives [73].

Although individuals can be classified by traits and characteristic adaptations, “they often (if not generally) communicate information about themselves differently than a simple nomothetic classification on these characteristics” (p. 15; [74]). Narratives are more contextually specific than the other layers and may provide explanations for why two people from different cultures, who are similar in traits and characteristics, may behave in different ways or find themselves in different circumstances. Research shows that people’s narrative techniques (including their use of tone, coherence, themes, and complexity) vary in unique ways that aren’t strongly reflected in other layers of analysis [75]. This suggests that examining narratives reveals important individual differences that might otherwise go unnoticed.

### Defining culture

It is difficult to capture the complexity and fluidity of culture in a single definition [76]; however, a general definition can be gleaned from common elements across the scholarly literature. At the core of almost every representation of culture in psychology is the shared behavioral and cognitive norms of members of a distinct group that are different from those shared by other groups [77]. These norms, often referred to as shared knowledge and meanings (see [76,78]), frame the perceptions, beliefs, understandings, communications, and behaviors of groups [78,79]. Culture forms around useful ideas (such as how to make a tool), which are passed through generations to inform collective knowledge [80]. For this to occur, a shared location, time, and language are integral and differentiate one culture from another [80,81]. When shared knowledge is widely distributed and accepted, it informs social expectations and behaviors [76].

In cross-cultural psychology, the cultural group is most commonly a national group (though studies have also compared ethnic groups; [82]), suggesting that the extant cross-cultural literature is largely cross-national. Although ethnic groups clearly share unique systems of meanings, language, customs, and history, nations also share these systems [83]. This is, in part, because nations are typically built around an ethnic core, where the values, language, laws, attitudes, and customs of one ethnic group develop into the characteristics of the nation [83–86].

Ethnic and national groups, however, can be distinguished: although nations are defined by geographic boundaries, ethnic groups do not require these and may even lack shared territory [84,85]. National characteristics are most often built around those of the ethnic majority group [83,87], resulting in circumstances where national and ethnic cultures overlap for members of the ethnic majority but not for ethnic minority groups [88,89]. Hence, a comprehensive review of personality assessment across cultures should include cross-national and cross-ethnic research. As such, for the purposes of this review and where possible, we have chosen to define a cultural group as a national or ethnic entity.

### Review aims and structure

Culture and personality are thought to significantly influence one another (e.g., [5,90]). As such, our understanding of personality and individual differences is deepened when social and societal contexts are considered [91]. However, further research is needed to understand the nature of the culture-personality relationship [90]. Such research requires meaningful comparisons of personality across cultural groups, and in order to do this, we need to understand the biases and strengths inherent within personality instruments.

To contribute to this understanding, the current scoping review aims to provide a broad overview of the main models and instruments used in personality assessment across cultures. Given the challenges of exporting etic (universal) models to non-Western cultural groups discussed above, the review will also assess the evidence of the reliability, validity, and cross-cultural measurement invariance of emic-etic models. As previously discussed, McAdams’s three-layer model of personality offers an empirically supported framework for addressing the research questions. By organizing the relevant research under these layers, this review provides a clearer and more comprehensive understanding of cross-cultural personality assessment that brings together evidence of the reliability, validity, and cross-cultural measurement invariance of models of traits, characteristic adaptations, and life-narratives.

Primary Research Question:

1. Can personality be assessed effectively across different cultures?

Secondary Research Questions:

1. Which models of personality are useful for personality assessment across cultures?
2. Which measures of personality can provide valid assessment of personality across cultures?
3. Which measures of personality can provide valid assessment of personality within specific cultures?

## Materials and methods

Reviewed research focused principally on a quantitative and nomothetic understanding of personality assessment, characterized by personality frameworks that apply across groups of people. No protocol was registered prior to the review; however, all methods and results are shown below without exception.

### Method

We reviewed the literature using a scoping review methodology [92] to provide insight into each research question. A scoping review was appropriate for this project given the breadth, complexity, and heterogeneity of the relevant literature [92,93]. Unlike systematic reviews, which focus on answering very specific questions based on relatively homogeneous and narrow research, scoping reviews excel at flexibly investigating complex topics. This scoping review provides a synthesized report on the models, methods, and available evidence regarding cross-cultural personality assessment.

### Search strategy

The review focused on relevant research published within the last 20 years identified by searching the following scientific databases: PsycInfo, Scopus, Google Scholar, Web of Science, and PubMed. Further research was identified through leading international (Tier 1) journals in personality and assessment journals (e.g., *the Journal of Personality Assessment, Assessment*, *Psychological Assessment*) and via reference lists and relevant previous literature reviews. The literature search was supplemented with articles from the University of Hong Kong database, which includes articles that have used the CPAI. Database searches occurred between January and the 5th of June 2024.

Our search strategy was conducted in two phases. In the preliminary phase, we used broad personality-related terms to identify the primary personality measures relevant to our review. After identifying the key frameworks, we refined our personality search terms to focus specifically on these frameworks across all three personality layers.

The final search strategy used three blocks of search terms: (1) personality, (2) assessment, and (3) culture (see Table 1). To ensure comprehensive coverage, we ran multiple search combinations:

**Table 1:** Search Term Blocks.

Search Term Block	Search terms
Block 1: Personality	
Preliminary search	personality OR “personality trait*” OR “characteristic adaptation*” OR “dispositional trait*” OR “life narrative*” OR “trait taxonom*” OR “personality model”
Layer 1 only	“Big Five traits” OR “Big Five inventory” OR “NEO PI-R” OR “mini IPIP” OR “NEO Personality Inventory Revised” OR “neo-pi” OR “hexaco” OR “Cross-cultural (Chinese) Personality Assessment Inventory” OR cpai OR “Chinese Personality Assessment Inventory” OR “South African Personality Inventory” OR “sapi”
Layer 2 only	“characteristic adaptation” OR “moral value” OR “Schwartz value” OR “personal value” OR “filial piety” OR “social axiom” OR “ism” OR “Survey of Dictionary-Based Isms”
Layer 3 only	“personality” OR “personality layer 3” OR “personality level 3” OR “narrative identity” OR “McAdams layer 3” OR “McAdams level 3”
Block 2: Assessment	
Layer 1 and 2	“personality assessment” OR “inventory” OR “questionnaire” OR “scale” OR “measure”
Layer 3 only	“Life Story Interview” OR “self-defining memory prompt” OR “autobiographical scenes” OR “life stor*” OR “self defining memories” OR “autobiographical memories”
Block 3: Culture	
Layer 1, 2, and 3	“cross-cultur*” OR “cross national”
**Search order**	**Combined search term blocks**
First	Block 1: preliminary search “AND” Block 2: Layer 1 and 2 “AND” Block 3
Second	Block 1: Layer 1 and 2 “AND” Block 2: Layer 1 and 2 “AND” Block 3
Third	Block 1: Layer 2 only “AND” Block 2: Layer 1 and 2 “AND” Block 3
Fourth	Block 1: Layer 3 only “AND” Block 2: Layer 3 only “AND” Block 3
Fifth	Mixed combination searches: e.g., Block 1: Layer 2 only “AND” Block 2: Layer 1 and 2

Limits = English Language, Year ≥ 2003.

- Searches using all three blocks together
- Targeted searches for layer 3 papers using only layer 3-specific terms in blocks 1 and 2
- Various combinations excluding one block at a time

The specific search syntax was adapted to meet each database’s requirements.

### Inclusion criteria

Each original study, review, and book chapter identified during the literature search was screened by one reviewer based on its abstract, title, and keywords. Extraction, screening, and evaluation were conducted using the Covidence online review platform [94], which also aided in the removal of duplicate publications. The review focused on research that was applicable to cross-cultural personality assessment, encompassing both universal models and measures used across cultures, as well as non-Western indigenous models. Emphasizing the inclusion of non-Western approaches is particularly important to advance the knowledge of cross-cultural personality assessment, given the under-representation of non-Western perspectives in prevailing personality models.

Inclusion criteria were:

- Published in English.
- Focused on personality and culture as defined above.
- Published within the last 20 years.
- Included the psychometric analysis or review of personality models and measures. Specifically, the assessment of the validity and reliability of personality measures, with a focus on comparisons across two or more cultures where possible, or within a non-Western culture (except where the measure was not developed in a Western country).

Where no articles including cross-cultural comparisons were found, articles with Western samples are included to provide a base level comparison of the instruments validity and reliability in different cultures.

### Data collection

An Excel spreadsheet was developed to collect results for the psychometric properties of the personality instrument(s) utilized in the reviewed articles (see S1 File). Data on the psychometric properties of the instruments was extracted based on the measurement outcomes defined by the COnsensus-based Standards for the selection of health Measurement Instruments (COSMIN; [95–97]). Extracted data was organized into: internal consistency, test-retest reliability, inter-rater reliability, content validity, structural validity, convergent/discriminant validity, incremental validity, criterion validity, and cross-cultural measurement invariance (configural, metric, and scalar). Beyond psychometric properties, data regarding the following instrument characteristics were collected: instrument name, article citation, and scale version, subscales or number of items used. Sample characteristics including number of samples, sample size, culture, and language that the instrument was administered in were also extracted. As Layer 3 is not measured using traditional personality instruments, data regarding psychometric properties could not be extracted in the same manner as articles collected under Layers 1 and 2. Instead, for Layer 3 models of personality, data regarding the instrument, aims, method, sample (number, size, culture), language and translation method (where possible), and cultural differences was extracted. A single reviewer extracted data from all 233 articles. The four person reviewer team met weekly to discuss and review the data extraction progress.

### COSMIN measurement ratings

The COSMIN criteria for psychometric properties was used to compare the psychometric quality of the scales included in this review. The COSMIN criteria have demonstrated strong rigor in systematic comparisons of non-clinical psychological instruments, such as of antisocial personality (known as the Dark Tetrad), attachment, and violence against children [98–101].

To guide the implementation of COSMIN ratings for psychometric properties (see Table 2), we used a modified version of Prinsen et al.’s [96] criteria, as applied by Welsh et al. [101] for the assessment of antisocial (Dark Tetrad) scales because of its relationship with personality assessment. Where necessary, the COSMIN criteria were modified to improve its applicability to personality models. The reviewed scales were given ratings for reliability, validity, and cross-cultural measurement invariance, based on pre-defined criteria. The COSMIN criteria for psychometrics are evaluated as ‘sufficient’, ‘indeterminant’ or ‘insufficient’ [96]; however, an additional set of ratings, were used in this review to reflect the more conservative cut-offs often used in personality psychology. Specifically, the current review rated the psychometric properties of personality instruments as ‘high’, ‘moderate’, ‘low’, or ‘unknown’ based on these cut-offs (see Table 2). Raw data from articles collected throughout the review are summarized at the end of each Layer subsection in the Results below. Where applicable, we provided an overall rating for each scale, along with recommendations for use. Ratings were based on a calculated average when multiple psychometric data were reported (e.g., internal consistency for an instrument with multiple subscales).

**Table 2:** COSMIN Criteria for Measurement Properties.

Measurement property	Rating	Criteria
Reliability		
Internal consistency^a^	High	Internal reliability coefficient(s) ≥.80 for each scale and subscales (Cronbach’s alpha and/or McDonald’s omega).
	Moderate	Internal reliability coefficient(s) ≥.70 for each scale and subscales (Cronbach’s alpha and/or McDonald’s omega).
	Low	Internal reliability coefficient(s) <.70 for each scale and subscales (Cronbach’s alpha and/or McDonald’s omega).
	Unknown	Internal reliability coefficient(s) not assessed or reported.
Test-retest	High	Correlations ≥ .70, timeframe appropriateness ≥ 5 weeks.
	Moderate	Correlations ≥ .70, timeframe appropriateness < 5 weeks.
	Low	Correlations ≤ .70, regardless of timeframe
	Unknown	Test-retest not assessed or reported.
Inter-rater^b^	High	ICC or weighted Cohen’s Kappa > .80.
	Moderate	ICC or weighted Cohen’s Kappa > .60.
	Low	ICC or weighted Cohen’s Kappa ≤ .60.
	Unknown	ICC or weighted Cohen’s Kappa not assessed or reported.
Construct Validity		
Content	High	*Scale development* involves evidence of the use of the following: • use of existing scales (where possible); • theoretical models; • expert consultation; • factor analysis (exploratory factor analysis and/or confirmatory factor analysis), principal components analysis, or item response theory; • replication of empirical findings (supported by additional studies). *Scale translation*: where scale translation is required: • a multiple translation method, such as the back-translation method [102] or double-translation procedure [15], is adhered to. • relevant experts are involved. • expert panel or independent translator review. • pilot testing.
	Moderate	Evidence of some (but not all) of the above.
	Low	Few or no scale development methods were evidenced or not well-supported.
	Unknown	Content validity not assessed or reported.
Structural^c^		
	High	CFA: • CFI or TLI or comparable measure > .95, • RMSEA < .06, • SRMR < .08.
	Moderate	CFI or TLI or comparable measure > .90 (other cut-offs the same).
	Low	CFI or TLI or comparable measure > .85 (other cut-offs the same).
	Unknown	Structural validity not assessed or reported.
Convergent	High	Correlation with established, comparable external variable ≥ .70.
	Moderate	Correlation with established, comparable external variable ≥ .40.
	Low	Correlation with established, comparable external variable ≥ .20.
	Unknown	Convergent validity not assessed or reported.
Incremental	High	The scale predicts an associated outcome over and above comparable scales, ∆*R*^2^ ≥ .15.
	Moderate	The scale predicts an associated outcome over and above comparable scales, ∆*R*^2^ ≥ .10.
	Low	The scale predicts an associated outcome over and above comparable scales, ∆*R*^2^ < .10.
	Unknown	Incremental validity not assessed or reported.
Criterion		
	High	Correlation with theoretically related external variable ≥ .40.
	Moderate	Correlation with theoretically related external variable ≥ .20.
	Low	Correlation with theoretically related external variable ≥ .10.
	Unknown	Convergent validity not assessed or reported.

ICC, intraclass correlation coefficient; EFA, exploratory factor analysis; CFA, confirmatory factor analysis; PCA, principal component analysis; CFI, comparative fit index; TLI, Tucker–Lewis index; RMSEA, root mean square error of approximation; SRMR, standardized root mean square residual.

^a^Prinsen et al.’s [96] criteria specify Cronbach’s alpha(s) ≥ 0.70 for a ‘sufficient evidence’ rating. This was increased to ≥ 0.80 for a ‘high’ rating in this review to reflect more conservative guidelines [103].

^b^Prinsen et al.’s [96] criteria specify ICC or weighted Kappa ≥ 0.70 for a ‘sufficient evidence’ rating. This was increased to ≥ 0.80 for a ‘high’ rating in this review to reflect more conservative guidelines.

^c^Structural validity cutoffs specified by Hu & Bentler [104].

## Results

### Overview

**Fig 1:**
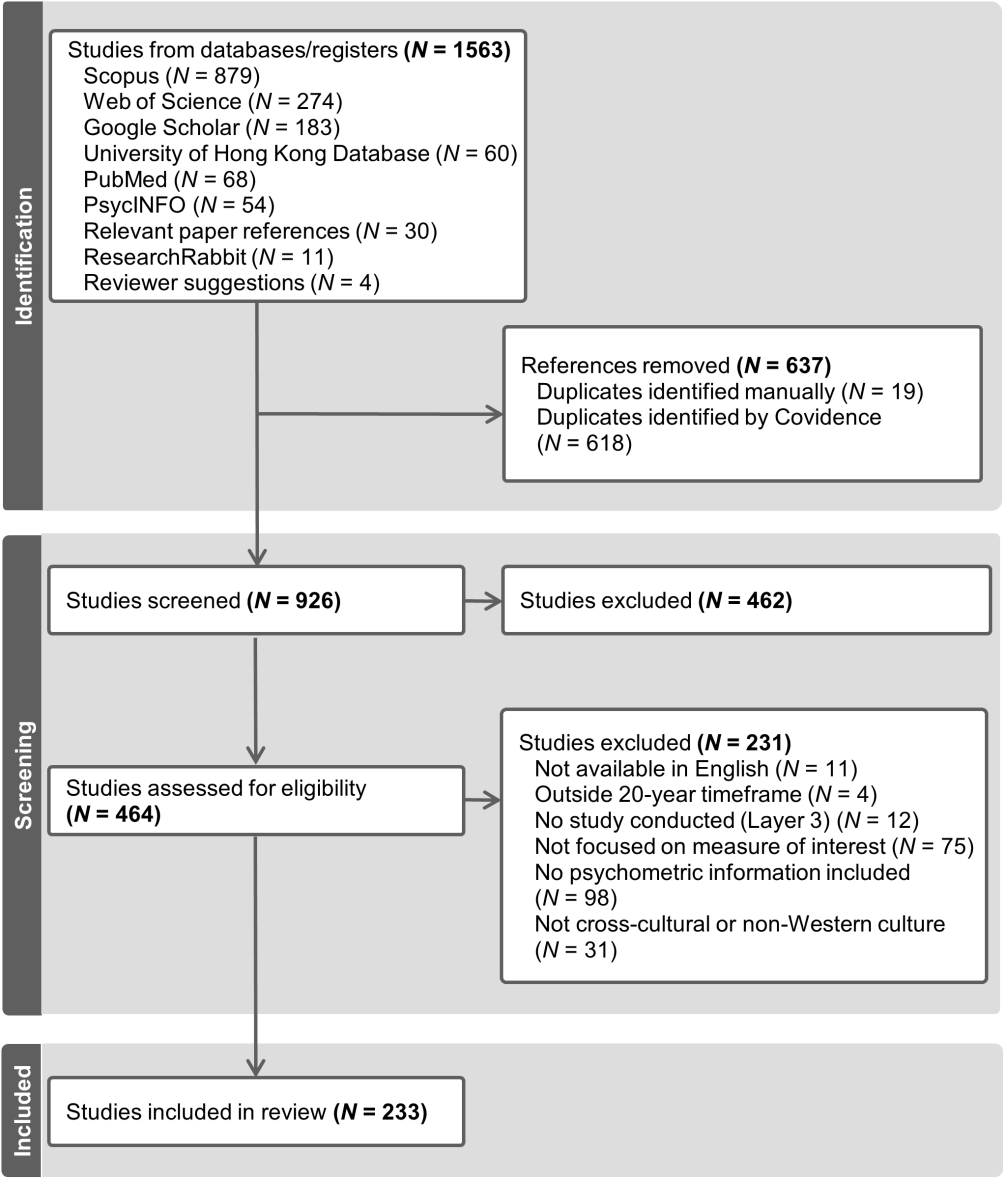
PRISMA Search and Selection Flow Chart.

The literature search identified 1563 articles, from which 637 duplicates were removed. The screening process removed 462 additional articles, and a further 231 articles were excluded after full-text eligibility checks, resulting in 233 articles for analysis. Fig 1 presents the PRISMA [105] flow chart of the identification, screening, and selection of articles. Of the 233 articles reviewed, 138 publications utilized personality trait instruments, 72 utilized models of characteristic adaptations, and 28 explored personality through life narrative instruments. Some articles utilized multiple instruments of personality.

### Layer 1: Trait models

Given the large number of instruments that are utilized to measure personality traits, this scoping review was restricted to include the most used trait models to avoid the scope becoming too large. Maladaptive trait models were also outside the scope of this review. Our database searches identified the Big Five (*N* = 79) and HEXACO (*N* = 22) as the most used trait models. The preliminary searches identified few articles utilizing the Eysenck Personality Questionnaire (*N* = 13) or Cattell’s 16PF Questionnaire (*N *= 2), which is supported by John et al. [11] who reported that the influence of Eysenck and Cattell’s models have decreased, whereas publications related to the Big Five have increased. The preliminary searches also identified 10 articles utilizing the Zuckerman–Kuhlman–Aluja Personality Questionnaire (ZKA-PQ; [106]) in cross-cultural comparisons or non-Western samples. The majority of identified articles using the ZKA-PQ (*n* = 6) were published from 2018, indicating use of this instrument in cross-cultural personality assessment may be increasing. If more relevant cross-cultural studies with this measure are conducted, future scoping reviews may want to include the ZKA-PQ as well; however, at this stage this review focused only on the most widely used scales.

The CPAI (*N =* 31) and SAPI (*N =* 10) models were identified as emic trait models. The Filipino indigenous Panukat ng Pagkataong Pilpino [107], Panukat ng Mga Katangian ng Personalidad [108], and Panukat ng Ugali at Pagkatao [109] scales were identified in one article [110]. However, limited psychometric information was reported and the culturally relevant traits appeared to be subsumed by the FFM [108]. Consequently, this review focused on the Big Five and HEXACO models for etic trait models and the CPAI and SAPI models for emic trait models.

### The Big Five model

The Big Five model [111,112] proposes a five-factor structure that describes personality, comprising the following traits: Openness to Experience (also known as Openness/Intellect), Conscientiousness, Extraversion, Agreeableness, and Neuroticism (also known as Emotional Stability). The most frequently used scales for assessing the Big Five include the NEO scales (*N* = 33; [113]), the Big Five Inventories (*N* = 34; BFI; [11,114,115]), and the International Personality Item Pool scales (*N* = 12; IPIP; [116,117]).

**The Big Five Inventory (BFI).** The 44-item BFI-44 [114] was the most commonly used BFI instrument (*N* = 18). It has been translated into over 29 languages, including Indonesian, Korean, Mandarin, Spanish, and Russian, and administered in 56 nations, including Australia, Fiji, Hong Kong, and the Philippines [118]. A large-scale, cross-cultural study showed that the five-factor structure generally replicated across several countries [118]. Nonetheless, some items, typically Openness items, demonstrated lower factor loading replicability among participants from South and Southeast Asian and African nations. The studies reported good internal consistency (e.g., [119–122]) and some supported the scale’s structural validity [123,124]. Metric invariance was supported for all factors except Neuroticism across 31 countries [125], and full scalar invariance was supported across European and Māori ethnic groups within New Zealand [124].

Other BFI instruments include the 60-item BFI-2 (*N = *5), the 30-item BFI-2-S (*N* = 1), the 15-item BFI-S (*N = *2), the 10-item BFI-10 (*N =* 4), the 50-item Big Five Personality Trait Short Questionnaire (*N* = 3; BFPTSQ), and Goldberg’s Big Five questionnaire (*N* = 1). Limited support for the cross-cultural utility of BFI-S was found, but the BFI-2 has reported good internal consistency in Japanese, English, Serbian, and Slovak [126–128], as well as good convergent validity in Slovak [126] and partial scalar invariance when comparing the U.S. and Serbia [127]. Nevertheless, a study in an Indian sample found the Kannada translation unsuitable for use [129]. The BFI-2-S demonstrated acceptable internal consistency, as well as good structural and convergent validity in samples from Germany and the U.S. [130]. Configural measurement invariance was supported, but only evidence of approximate scalar invariance was found. The BFI-10 demonstrated satisfactory reliability and validity in Germany and the U.S. [131], but its measurement invariance across countries has not been supported [132]. The BFPTSQ demonstrated good internal consistency in English, Spanish, and Dutch [133–135], but low structural validity in samples from Spain, Argentina, and America [133]. Configural invariance was supported across national groups and languages in samples from Spain, Argentina, and America, but only evidence of partial metric invariance was found. Goldberg’s Big Five questionnaire demonstrated moderate internal consistency in an Italian sample and high internal consistency in a Dutch sample, but only configural invariance was supported across the two samples [136].

One article reported the use of ant colony optimization (a meta-heuristic optimization procedure used to develop optimal short scales) to create a 15-item short form of the BFI-2 for use across France, Germany, Poland, Spain, and the U.S. [137]. The measure demonstrates good model fit and scalar measurement invariance, meaning that the structure was equivalent across cultures and statistically unbiased comparisons of personality profiles can be made. The scale’s lower internal consistency (average α = .63) is generally considered acceptable for brief measures.

**The NEO Personality Inventory Revised (NEO PI-R).** The NEO Personality Inventory Revised [113] and the NEO-PI-3 [138], which was revised slightly to improve item readability, are often discussed in conjunction with one another given their similarity. The NEO-PI-R/NEO-PI-3 were the most utilized measure of the Big Five (*N* = 24) articles reviewed. The NEO-PI-R/NEO-PI-3 have demonstrated evidence of strong internal consistency across 49 cultures [62,139,140]; however, coefficient alphas for Openness were low in India, Malaysia, Botswana, Ethiopia, and Uganda. The addition of 15 emic Russian personality items provided no additional information beyond the initial NEO-PI-R and principal components analysis indicated that the emic items were subsumed by other Big Five dimensions [141].

Evidence for measurement invariance varied across articles. Scalar invariance was supported across 23 cultures for all factors except Openness, for which only evidence of configural invariance was found [142]. Other studies reported evidence of scalar invariance for all five factors in Serbian and German samples [143], metric invariance for all five factors in nine French-speaking African countries and Switzerland [64], and partial scalar invariance in European and Asian American samples [144]. The shorter, 60-item NEO Five Factor Inventory (NEO-FFI) was evaluated in nine articles. The scale demonstrated evidence for moderate to high internal consistency across studies, though low alpha coefficients were reported for Openness in Chinese, Turkish-Dutch and Moroccan-Dutch, Iranian, Croatian, and Singaporean samples [145–150]. Moreover, no evidence that the structural validity or cross-cultural measurement invariance of the NEO-FFI had been tested was found in this review.

**The International Personality Item Pool (IPIP).** Use of measures from the International Personality Item Pool (IPIP) was identified in 12 articles. The IPIP provides measures of various lengths, targeting many constructs, but is primarily concerned with the measurement of the Big Five personality traits [116]. The 50-item IPIP reflecting Goldberg’s [151] markers for the Big Five factor structure was the most commonly utilized measure (*N* = 5) and reported good internal consistency in Croatian, Chinese, British, Cypriot, Australian, and Indian samples [152–155]. Principal components analysis and congruency coefficients suggest that the five-factor structure was replicated in Mandarin and Croatian translations of the scale [154,155]. Nevertheless, no studies included in this review conducted an analysis of the cross-cultural measurement invariance.

The 20-item Mini IPIP [156], one of the shorter measures of the Big Five, was identified in three articles. The scale demonstrated lower internal consistency in a Ukrainian (average α = .61) and Malaysian (average α = .56) sample [157,158]. A study including samples from the USA and Chile reported support for the internal consistency, structural validity (with correlated uniqueness), and partial scalar invariance of the scale [159].

The IPIP-NEO-120 [117], a 120-item representation of the NEO-PI-R [113,160], was identified in four articles. The scale reported good internal consistency and evidence supporting its structural validity across 22 countries [161–163]. Cross-cultural scalar invariance was supported for the Neuroticism, Extraversion, and Conscientiousness factors, and metric invariance for the Agreeableness and Openness factors, across 12 countries [163]. The IPIP-NEO-300, a 300-item representation of the NEO-PI-R [113,160], was identified in one paper. However, no reliability or validity estimates were reported and measurement invariance was not supported across 10 countries [164].

### The HEXACO model

In the 22 articles utilizing the HEXACO model in this review, four used versions of the HEXACO Personality Inventory (HEXACO-PI) and 17 used versions of the HEXACO Personality Inventory Revised (HEXACO-PI-R; [38,165]). The HEXACO-PI demonstrated evidence of high internal consistency in Japanese, English, Dutch, and Italian speaking samples (e.g., [166–168]) and moderate internal consistency in a Spanish speaking sample [169]. The Extraversion and Conscientiousness subscales showed evidence of high convergent validity in Japanese, Canadian, Australian, and Dutch samples and the remaining subscales demonstrated a mixture of moderate to high convergent validity across these groups [165–167]. A single article reported evidence of the HEXACO-PI scale’s incremental validity over Big Five model measures, indicating that the additional Honesty-Humility factor may provide incremental validity beyond the Big Five traits [167]. We found no articles that assessed the cross-cultural measurement invariance of the HEXACO-PI scale.

The HEXACO-PI-R demonstrated evidence of high internal consistency in samples from Iran, Serbia, South Korea, the United Kingdom, the United States, Germany, and Mainland China [170–175]. Three cross-cultural studies reported combined sample coefficient alpha and omega values > .80; however, no information was provided on these values within individual cultural groups [176–178]. Evidence for the incremental validity of the HEXACO-PI-R was reported in a Serbian sample [174] and an Iranian sample [175]. Studies in these samples demonstrated that small changes in the amount of explained variance were found when compared to the Big Five measures. Principal components analysis supported the six-factor structure in six studies [38,173–175,177,179]. Confirmatory factor analysis found low evidence of structural validity in Oman and Thai samples and moderate evidence in Indian, Indonesian, and Romanian samples [180]. A large cross-cultural study supported the configural and metric invariance across translated versions of the 100-item HEXACO-PI-R, suggesting that the structure and meaning of the six factors are similar across languages [177]. Nevertheless, no support for scalar invariance was found, preventing meaningful comparison of mean-level trait scores across languages. Another cross-national study supported configural and metric invariance for the English version of the HEXACO-PI-R across 33 countries [176].

### Cross-Cultural (Chinese) Personality Assessment Inventory (CPAI)

Use of the CPAI model was identified in 31 articles, eight using the original CPAI measure [57], 20 using the refined CPAI-2 [56], and three using the CPAI-Adolescent scale (CPAI-A; [181] as cited in [61]). The full measure (Form A) contains 28 personality scales (Form B; 298-items), 12 clinical scales (Form C; 225-items), and three validity scales designed to assess participant response consistency and quality (used in conjunction with Forms B and C; 43-items). The personality scales reflect the four dimensions found in the CPAI model.

Studies that utilized the full CPAI reported moderate to high internal consistency in samples from Hong Kong and Mainland China [58,182,183] and low internal consistency in samples from the United States and Singapore [184,185]. A study using the English version in samples from Singapore and the USA indicated that the four factors could be replicated and that the Interpersonal Relatedness factor was distinct from the Big Five factors found in the NEO-FFI scale [184]. No studies assessing the cross-cultural measurement invariance of the CPAI were identified.

The CPAI-A demonstrated moderate internal consistency in a sample from Hong Kong [186] and high internal consistency in a sample from Mainland China [187]. The Harmony subscale from the Interpersonal Relatedness scale accounted for a small addition variance in life satisfaction beyond the universal personality scales (the CPAI factors that have been found to relate to the Big Five factors; [186]). No articles assessing measurement invariance or utilizing the CPAI-A outside Mandarin or Cantonese speaking populations were identified.

The CPAI-2 scale reported low internal consistency in Mainland Chinese samples and moderate internal consistency in Hong Kong samples [188]. Principal components analysis in one article supported the four-factor structure [56]. Two short-forms of the CPAI-2 Form B were developed in a single article [60]. In a Chinese sample, the 56-item CPAI demonstrated moderate internal consistency (average α = .79), where the 28-item CPAI demonstrated low internal consistency (average α = .68) in a Chinese sample [60]. The four-factor structure demonstrated acceptable fit for both scales (.90 < CFI < 0.95, and RMSEA < .06).

We identified six studies utilizing the CPAI model outside Cantonese- and Mandarin-speaking populations (namely those residing in Hong Kong, Mainland China, and Taiwan). Studies using English versions of all 28 personality scales reported low to moderate internal consistency in Singaporean, South African, and U.S. samples [59,189–191]. A study using only the Traditionalism vs. Modernity personality scale reported moderate internal consistency across Jewish, Arab, and Ethiopian Israeli samples [192]. Issues with English-translated items that involved negations, unfamiliar words, and concepts that had different connotations across cultural groups were reported in a study including South African samples [191]. Factor analysis did not support the four-factor structure in another South African sample [190]; however, a distinct Interpersonal Relatedness factor was found. We did not identify any articles assessing the cross-cultural measurement invariance of the CPAI-2.

### The South African Personality Inventory (SAPI)

The SAPI model was evaluated in 10 articles. Studies within South African populations demonstrated high internal consistency [67,191,193–196], strong support for the six-factor structure [68, 196], and scalar invariance across South African ethnocultural groups (Black, Colored, White, and Indian; [196]). Principal components analysis replicated the social-relational factors in “mainstream” and “immigrant” (“Western immigrants”, “Antillean, Surinamese, and Indonesian immigrants”, and “non-Western immigrants”) groups in a Dutch sample with moderate internal consistency [191]. The six-factor model was supported across ethnic groups in New Zealand European and Māori samples, demonstrating high internal consistency, metric invariance across the ethnic groups, and small incremental validity for predicting family orientation [124]. In a White South African sample, the positive social-relatedness scale also demonstrated incremental validity beyond the universal traits for predicting prosocial tendencies, expressed as altruistic helping and empathy [191].

### Summary of Layer 1

The NEO-PI-R/NEO-PI-3 measure was the most commonly utilized of the instruments identified in the 138 articles in Layer 1 (24/138). It demonstrated the highest cross-cultural measurement invariance and acceptable psychometric properties. These findings suggest that NEO-PI-R is the most useful trait model instrument for cross-cultural research. Table 3 presents the summarized reliability, validity, and measurement invariance ratings for each instrument (the raw data for each article can be found in S1 File).

**Table 3:** Reliability, Validity, and Cross-Cultural Applicability of Layer 1 Measures.

Measure	Reliability^a^			Validity^b^				MI^c^	Recommendations	
	1.	2.	3.	1.	2.	3.	4.		Rating	Comments
Big Five Model										
44-item Big Five Inventory (BFI-44)	H	U	M	H	M	M	U	Metric (across 31 cultures) Scalar (cross-ethnic groups in New Zealand)	Maybe	The BFI-44 has demonstrated acceptable psychometric properties and metric invariance across 31 countries. The large number of freely available translations makes the scale particularly useful. Scalar invariance has also been supported across ethnic groups in a culturally heterogeneous country, indicating its usefulness in cross-cultural research within countries. Consider using the BFI-44 if: − the population of interest is ethnically heterogenous; − the research seeks to understand personality within countries but not compare across them.
10-item Big Five Inventory (BFI-10)	H	L	H	H	L	M	U	No support	No	The lack of support for measurement invariance makes this scale unsuitable for cross-cultural research.
15-item Big Five Inventory (BFI-S)	U	U	L	U	U	U	U	Unknown	No	The lack of psychometric evidence and support for measurement invariance makes this scale unsuitable for cross-cultural research.
60-item Big Five Inventory (BFI-2)	M	U	M	H	M	H	U	Partial scalar (the U.S. & Serbia)	No	The BFI-2 has shown some utility in cross-cultural research and demonstrated acceptable psychometric properties. However, the limited cross-cultural research, increased number of items, and inability to replicate in India suggest that the BFI-44 is a better option for a BFI instrument in cross-cultural research.
30-item Big Five Inventory (BFI-2-S)	H	U	M	H	H	H	U	Scalar for N (approximate for all) Metric for E & C Configural for A & O (the U.S. & Germany)	No	For a short BFI measure, the BFI-2-ACO is recommended for cross-cultural comparisons.
15-item Ant Colony Optimisation Big Five Inventory (BFI-2-ACO)	U	U	L	H	M	H	U	Scalar	Maybe	The BFI-2-ACO scale offers a short (15-item version), scalar invariant version of the BFI-2 which may be useful if item parsimony is necessary. However, the existing cross-cultural research with this scale is limited and translations may be limited.
Big Five Personality Trait Short Questionnaire	M	U	H	H	M	M	U	Partial scalar (Argentina, Spain, and the U.S.)	No	For a short BFI measure, the BFI-2-ACO is recommended.
NEO Personality Inventory Revised (NEO-PI-R/ NEO-PI-3)	M	L	H	H	L	M	U	Scalar for all but O (across 23 cultures) Openness: Configural (across 23 cultures) Metric (10 French-speaking countries) Scalar (Serbia and Germany)	Yes	The NEO-PI-R demonstrates the highest cross-cultural measurement invariance and acceptable psychometric properties. The lack of support for scalar measurement invariance of the Openness facet may be expected given that the trait Openness may exist differently across cultures. This should be considered when assessing personality across cultures.
NEO Five Factor Inventory (NEO-FFI)	M	L	M	H	U	U	L	Unknown	No	The lack of evidence for measurement invariance makes this scale unsuitable for cross-cultural research.
20-item International Personality Item Pool (Mini-IPIP)	U	U	L	H	L	U	U	Partial scalar (the U.S. & Chile)	Maybe	The mini-IPIP may have utility in offering a freely available, very short measure of the Big Five with acceptable psychometric properties. The low internal consistency is expected with such a measure. A version of the scale that includes items for the HEXACO honesty-humility trait is available. Consider using the mini-IPIP only if item parsimony is required.
120-item International Personality Item Pool NEO (IPIP-NEO-120)	U	U	H	H	M	U	U	Scalar for N, E, & C Metric for A & O (across 12 cultures, English version only)	Maybe	The IPIP-NEO-120 offers a freely available broad-bandwidth instrument, akin to the NEO-PI-R. The IPIP-NEO-120 has also demonstrated higher level measurement invariance for the Openness facet; however, this is only for the English version. The IPIP-NEO-120 appears to be available in more languages than the NEO-PI-R.
50-item International Personality Item Pool	U	L	H	H	L	H	U	Unknown	No	The lack of evidence for measurement invariance makes this scale unsuitable for cross-cultural research.
HEXACO										
HEXACO Personality Inventory (HEXACO-PI)	U	L	H	H	L	M	H	Unknown	No	Use HEXACO-PI-R.
HEXACO Personality Inventory Revised (HEXACO-PI-R)	M	L	H	H	M	M	L	Metric (across 33 cultures and 16 languages)	Maybe	The HEXACO-PI-R may provide the most comprehensive etic scale for personality assessment. It demonstrates the strongest psychometric properties, and the additional honesty-humility trait provides some incremental validity beyond the Big Five traits. The lack of support for scalar metric invariance suggests that personality scores cannot be meaningfully compared across cultures. As such, this scale may not be useful for comparing the mean levels of personality across cultures.
Cross-Cultural (Chinese) Personality Assessment Inventory (CPAI)										
CPAI	L	U	L	H	L	L	U	No level supported	No	Use of the refined CPAI-2 is recommended over the CPAI.
CPAI-2	U	U	M	M	L	M	H	Unknown ^d^	Maybe	If one is able to gain access to the scale, the CPAI-2 may be useful in assessing personality in East Asian countries. The focus on traditional Confucian values, limited use in existing cross-cultural research, lack of transparency, and lack of translations limit the use of the CPAI-2 in cross-cultural personality assessment. Consider using the CPAI-2 if: − the traditional cultural elements of the CPAI model are relevant to the research or population; − external variables related to the interpersonal traits are of interest; − the research is interested in comparing the traditional aspects of the CPAI across cultures.
South African Personality Inventory (SAPI)										
SAPI	U	L	H	H	H	M	H	Scalar (across ethnic groups in South Africa) Metric (across ethnic groups in New Zealand)	Maybe	The SAPI can provide a comprehensive assessment of personality including interpersonal related traits. The SAPI has demonstrated stronger psychometric properties than the CPAI and may have greater utility in cultures that do not relate to traditional Chinese (or East Asian) values. The lack of use in existing cross-cultural research and translations reduce its utility in cross-cultural personality assessment. The use of the SAPI is restricted by its copyright and the limited translations. Consider using the SAPI if: − external variables related to the interpersonal traits are of interest; − the population of interest is ethnically heterogenous; − the population of interest is not only East Asian; − the research seeks to understand personality within countries and not compare across them; − have the capacity to translate the scale into languages of interest.

H, High; M, Moderate; L, Low; U, Unknown; Maybe, use is recommended, but with understanding of the instrument’s limitations. O, Openness; C, Conscientiousness; E, Extraversion; A, Agreeableness; N, Neuroticism.

^a^Reliability: 1. Test-retest; 2. Inter-rater; 3. Internal consistency.

^b^Validity: 1. Content; 2. Structural; 3. Convergent; 4. Incremental.

^c^The highest level of measurement invariance achieved (lowest = configural, middle = metric, highest = scalar).

^d^Scalar MI across Euro-Canadian and Han Chinese samples for the Somatization clinical scale only has been supported through ordinal regression tests; although this suggests that items are interpreted in the similar way, this method is not as robust as standard measurement invariance test [197].

### Layer 2: Characteristic adaptations

The preliminary literature searches identified Schwartz’s [72] value theory as the most commonly used model of values (*N* = 34). Additionally, moral foundations (*N* = 10; [198–201]) and filial piety (*N* = 13; [202]) were identified as relevant components of values. Broad frameworks used to measure beliefs included social axioms (*N* = 10; [70]) and “isms” (*N* = 9; [203]). Overall, 72 articles using measures of characteristic adaptations were identified.

### Schwartz’s value theory

Schwartz’s value theory posits the existence of 19 non-discrete, basic human values (e.g., Tradition, Face, Hedonism) that form a circular continuum (see, [204]). Five instruments were utilized to measure Schwartz’s values across 34 articles: the 56-item or 57-item Schwartz Value Survey (*N* = 15; SVS; [72]), the Short Schwartz Value Survey (*N* = 3; SSVS; [205]), and the 40-item (*N* = 8) or 21-item (*N* = 12) Portrait Values Questionnaire (PVQ; [206]). The PVQ measures performed the best, demonstrating evidence of high internal consistency in South African and Austrian samples [207], as well as two Chinese samples [208,209]. Moderate evidence of internal consistency was also reported in samples from Isreal [210] and Germany [211]. Two cross-cultural studies reported moderate average internal consistency [212,213], and one reported high to moderate across 17 countries (Openness to Change α = .77; Conservation α = .75; Self-Transcendence α = .80; Self-Enhancement α = .83; [214]). The PVQ21 demonstrated full metric invariance for all scales, and partial scalar invariance for the Self-Transcendence, Self-Enhancement, and Conservation scales, across China, Germany, and Russia [215]. The PVQ-40 demonstrated only partial metric measurement invariance across 14 countries [213]. The PVQ21 has established low average intraclass correlations (.04) across 19 countries [216], which indicates that only 4% of differences in self-rated values were accounted for by the country of origin. This suggests that Schwartz’s values are consistent across cultures.

### Moral values

The Moral Foundations Questionnaire (MFQ), which was developed to measure the five moral values (Purity, Ingroup Loyalty, Authority, Harm, and Fairness), was evaluated in nine articles. The original study supported the proposed five-factor framework in large Western, primarily U.S., samples [198]. Although the measure appears to work well in Western societies, limited evidence exists for its structural validity outside Western cultures. For example, the five-factor model of the MFQ failed to converge in a study of 27 countries (conducted in the countries’ official languages; [217]). Further, measurement invariance was not supported in a study including samples from Iran and the U.S. [218]. This suggests that the findings obtained by Graham et al. [198] could not be replicated across many cultures. The Persian translation of the MFQ, however, demonstrated small incremental validity over the 10-Item Personality Inventory (a brief measure of the Big Five domains; [219]) when predicting belief in COVID-19 conspiracy theories in an Iranian sample [220].

A new version of the Moral Foundations Questionnaire-2 (MFQ-2) was developed in 2023, which measures additional values (splitting fairness into equality and proportionality), proposing an alternative six-factor model [218]. This study used the MFQ-2 in 19 countries and across diverse languages (Spanish, Japanese, English, Arabic, French, and Russian) to demonstrate better psychometric properties than the original MFQ. The MFQ-2 demonstrated moderate incremental validity over the MFQ-1 when predicting various ideologies and values, including collectivism, religiosity, left-wing authoritarianism, and empathy. Scalar measurement invariance was supported for all factors except purity, where most non-invariance was reported to be due to unique item intercepts in the Argentinian and Chilian samples.

### Filial piety

Measures of filial piety, a set of Confucian-based traditional values, practices, and moral norms regarding how people should behave towards their parents and ancestors [221], were reported in 13 articles. The dual filial piety model [202] was measured using the 16-item Dual Filial Piety scale (*N* = 9; DFPS; [202]), while a single article [222] utilized a 10-item scale. The DFPS demonstrated high internal consistency in samples from Australia, Singapore, Indonesia, Yemen, Poland, Hong Kong, and Mainland China [222–228], and moderate internal consistency in a sample from Malaysia [229]. It also demonstrated acceptable structural validity in Australia, Singapore, and Poland [227,228]. No articles investigating the cross-cultural measurement invariance of the DFPS were identified.

The 25-item Filial Behaviour scale (FBS; [230,231]) was evaluated in two articles. The scale demonstrated moderate convergent validity with filial piety attitudes and similar relationships with the Schwartz Value Survey and Social Axioms Survey (discussed below), indicating that it is measuring a similar construct to filial piety attitudes [231]. Additionally, the FBS demonstrated high internal consistency and structural validity in Hong Kong, Mainland China, Italy, Malaysia, and the U.S. [230,231]. Partial scalar invariance was also supported across samples from the U.S., Italy, and Malaysia [230]. It should be noted, however, that a shorter 10-item version of the scale performed better in these samples than the original 25-item version.

Filial piety was also assessed in two articles using the 10-item Contemporary Filial Piety scale (*N = *2; CFPS; [232]) and the 15-item Three-Dimensional Filial Piety scale (*N* = 1; TDFPS; [233]). The CFPS demonstrated high internal consistency and structural validity across samples from Hong Kong [232,234]. Nevertheless, neither article assessed measurement invariance. The TDFPS was used in a sample from China and demonstrated high internal consistency, test-retest reliability, and structural validity [233]. Correlations between the TDFPS and other measures of filial piety (the DFPS and CFPS), however, indicated a low COSMIN rating for convergent validity, and measurement invariance was not tested.

### Social axioms

Social axioms are general beliefs about how the world works [235], including beliefs about oneself, the physical and social environment, and the spiritual world [70]. They have a five-factor structure containing Social Complexity (human behaviour varies across situations and there are numerous ways to achieve an outcome), Religiosity (belief in a supreme being influencing the world and the positivity of religious practice), Reward for Application (effort, knowledge, and careful planning will lead to positive results), Social Cynicism (a negative view of human nature), and Fate Control (the belief that life events are predetermined and influenced by destiny; [236]). Social axioms are typically measured using the Social Axioms Survey (SAS), which was identified in 10 articles. The scale demonstrated average low internal consistency across 33 countries [237] and partial metric measurement invariance across the native languages of 23 countries [238]. The five-factor structure was supported at the individual and cultural level across 33 countries, including Australia, China, Hong Kong, the Philippines, and Russia [239]. Intraclass correlation coefficients (ICC) have suggested that across nine world regions (Southeast Asia, sub-Saharan Africa, South Asia, Latin America, Middle East/North Africa, West Europe, East Europe, Anglo and East Asia) the most pronounced cross-cultural differences (i.e., variance explained by the world region a person is from) are in religiosity beliefs (32% of variance; [239]). Western Europe reported the lowest average religiosity scores, whereas Southeast Asia reported the highest. The SAS, particularly the Fate Control dimension, demonstrated incremental validity beyond the CPAI-2 scale and a measure of self-esteem when predicting life satisfaction in a sample from Mainland China [240]. It also demonstrated some incremental validity over the SVS when predicting vocational interest, styles of conflict resolution, and coping style in a sample university students from Hong Kong [235].

### Isms

The term “isms” is given to a system of social attitudes and beliefs (e.g., traditionalism, materialism, Taoism, ethnocentrism, rationalism; [69,203]). They have a five-factor structure comprising Tradition-oriented Religiousness (belief in and support for conventional and traditional forms of religion), Unmitigated Self-interest (justification of forms of self-interest), Communal Rationalism (support for a nation, individual freedom, and the use of reason), Subject Spirituality (value of spiritual or paranormal experiences), and Inequality-Aversion (egalitarianism). Isms were measured using the 46-item (*N = *8) and 28-item (*N =* 1) Survey of Dictionary-Based Isms (SDI; [69]). The SDI has demonstrated acceptable reliability and validity; however, this review found no evidence that the instrument’s cross-cultural measurement invariance has been tested. Research with the SDI mostly occurred in the U.S. and within Eastern European countries, such as Serbia and Romania [241–243]. Nevertheless, SDI was also used in one large cross-cultural study, where it appeared to achieve lower levels of internal consistency [237].

### Summary of Layer 2

Table 4 presents the summarized ratings for each Layer 2 instrument. The PVQ21 demonstrated acceptable psychometric properties and support for partial scalar invariance of the higher-order Self-Transcendence, Self-Enhancement, And Conservation values, and metric invariance for the Openness to Change value. Although a new measure, the MFQ2 may prove useful for assessing and comparing moral values across cultures, with caution being exercised when using the Purity scale. The culture-specific value of filial piety has rarely been used outside Chinese-speaking countries. Nonetheless, Filial Behaviour Scale shows promise for assessing filial piety more globally.

**Table 4:** Reliability, Validity, and Cross-Cultural Applicability of Layer 2 Measures.

Measure	Reliability^a^			Validity^b^				MI^c^	Recommendations	
	1.	2.	3.	1.	2.	3.	4.		Rating	Comments
Personal Values										
Schwartz Value Survey	U	U	M	H	M	M	U	Unknown	No	The lack of evidence for measurement invariance makes this scale unsuitable for cross-cultural research.
40-item Portrait Values Questionnaire (PVQ40)	U	U	M	H	M	U	U	Partial metric	No	Use of the PVQ21 is suggested.
21-item Portrait Values Questionnaire (PVQ21)	U	U	M	H	H	U	U	Partial scalar for S-T, S-E, and Conservation Metric for Openness	Yes	The PVQ21 is the recommended scale for assessing personal values. It demonstrates acceptable psychometric properties and support for measurement invariance of higher-order values. The scale is freely available in at least 39 languages.
Moral Values										
Moral Foundations Questionnaire (MFQ)	H	U	M	H	L	U	L	No support	No	Use is not recommended.
Moral Foundations Questionnaire 2 (MFQ-2)	U	U	H	H	H	H	M	Scalar supported for all except purity	Yes	The MFQ-2 could be useful in assessing moral foundations. It demonstrates strong psychometric properties. The scale is new, with only one cross-cultural study – scalar measurement invariance was supported for all but purity, suggesting that group-level means may not be comparable across purity items (particularly in Argentina and Chile).
Filial Piety										
16-Item Dual Filial Piety Belief Scale (DFPB)	U	U	H	H	M	M	U	Unknown	Maybe	Acceptable structural replication of the DFPB scale in Singapore and Australia suggest that the scale may have some utility in assessing filial piety outside Chinese-speaking societies. However, the lack of support for measurement invariance makes it unclear if cross-cultural comparisons can be made.
Contemporary Filial Piety Scale	U	U	H	H	H	U	U	Unknown	No	The lack of psychometric evidence and support for measurement invariance makes this scale unsuitable for cross-cultural research.
Three-Dimensional Filial Piety Scale (3DFP)	H	U	H	H	H	L	U	Unknown	No	The 3DFP demonstrates the strongest psychometric properties. The scale, however, is new and its utilization in research is limited. Additionally, the lack of evidence for measurement invariance means caution is needed when using the scale and comparison of filial piety across countries is not recommended.
Filial Behaviour Scale (FBS)	U	U	H	H	H	M	U	Partial scalar	Maybe	The FBS taps into the behavioral manifestations of filial piety. It demonstrates good psychometric properties and partial scalar invariance across the U.S., Italy, and Malaysia, indicating potential utility in cross-cultural research. The evidence, however, is limited.
Social Axioms										
Social Axiom Survey	U	U	L	M	M	M	L	Partial metric	Maybe	The SAS demonstrates acceptable psychometric properties; however, cross-cultural studies report lower internal consistencies across countries. The support for the five-factor structure and partial metric invariance across countries suggest that the SAS may be used to look at country data individually, but meaningful cross-country comparisons are not possible.
“Isms”										
Survey of Dictionary-Based Isms, Version B	U	M	M	H	U	M	U	Unknown	Maybe	The SDI demonstrates acceptable psychometric properties. However, the lack of support for measurement invariance and the existence of culture-specific isms suggests high caution is warranted when using this measure in cross-cultural research.

H, High; M, Moderate; L, Low; U, Unknown; Maybe, use is recommended, but with understanding of the instrument’s limitations.

^a^Reliability: 1. Test-retest; 2. Inter-rater; 3. Internal consistency.

^b^Validity: 1. Content; 2. Structural; 3. Convergent; 4. Incremental.

^c^The highest level of measurement invariance (MI) achieved (lowest = configural, middle = metric, highest = scalar).

### Layer 3: Life narratives

Twenty-eight articles using life narrative approaches in cross-cultural or non-Western studies were identified. Across the articles, five specific analytic methods were implemented: the Life Story Interview (*N* = 3; LSI, [244]); Singer’s self-defining memory task (*N* = 8; [245]); the Peak-Experience Interview (*N* = 6; [246,247]), the Autobiographical Memory Test (*N* = 3; AMT, [248]); and, the Thinking About Life Experiences scale (*N* = 5; TALE, [249]). A further four articles used thematic analysis of interviews designed to prompt participants to provide descriptions of key scenes (e.g., life high points and low points). It is important to note that this layer of personality has not been investigated with traditional personality measures. Accordingly, this review was not able to use traditional psychometric indices to evaluate the cross-cultural integrity and/or applicability of this Layer. Instead, we report on how narrative identity has been assessed and the cultural differences and similarities reported across studies.

### Life story interview

Three articles utilizing the LSI followed a specific interview structure and coding scheme including topic, valence, coherence, subjective perspective, relationships, and identity connections. Two articles reported good inter-rater reliability among coders (average reported κ = .80, [250]; average reported κ = .83, [251]). The narrative coherence of intergenerational narratives was reported to uniquely contribute to well-being outcomes in Chinese, Māori, and European New Zealand adolescents [250]. Weak correlations between Big Five personality traits and the narrative constructs of causal coherence (drawing connections between past events and the person they are presently) and thematic coherence (integrating past events to establish a central theme for one’s life story) were also reported in Chinese, Māori, and European New Zealand adolescents [251]. A study exploring personal continuity (a sense of self that persists over time and situation) in American children of immigrants (Cambodia, Greece, India, Korea, Mexico, Puerto Rico, Samoa, and the Philippines) reported that all participants related their identity to the group-level narrative of leaving what is familiar to start again with nothing and working hard to achieve the “American Dream” [252]. Participants also related their identity to the historical narratives of their parents’ country of origin and the experience of being an ethnic minority in America.

### Self-defining memory task

The self-defining memory (SDM) task explores stories that participants consider important, enduring, and highly emotional [245]. Moderate to high inter-rater reliability was reported across six articles (average reported κ = .83, [253]; average reported κ = .83, [254]; average reported κ = .81, [255]; average reported κ = .81, [256]; average reported κ = .73, [257]; average reported ICC = .77, [258]). SDMs reported moderate to high convergent validity with personality traits across cultures, where frequency of SDM recall and Extraversion were positively correlated (*r *= .41, *p* < .01) in a Chinese sample, and Openness correlated with both positive memory affect (*r *= −.28, *p* < .05) and negative memory affect (*r *= .33, *p* < .05) in an American sample [259]. Cross-cultural studies reported differences in the SDMs recalled across groups. For example, Chinese college students were more likely to incorporate academic stress, guilt, and shame into their SDM than American college students [259,260]. Trauma survivors with PTSD reported more SDMs related to trauma than those without PTSD in a sample from independent cultures (focus on individual autonomy), but not in a sample from interdependent cultures (focus on social context; [255]). SDMs also reflected similarities in narrative identity across ethnic or cultural groups in the United States, where negative bicultural memories told with positive resolutions were predictive of higher bicultural identity integration [258]. A longitudinal study conducted with an ethnically diverse group of Americans (White, Asian American, Latino, Filipino, Black, and Arabic/Middle Eastern) found that experiences of prejudice, discrimination, or racism, and positive experiences of connection with their ethnic or cultural group altered people’s narrative identities [256].

### Peak-experience interview

Peak-experiences are memories of particularly wonderful or joyful experiences [246,247]. One article reported high inter-rater reliability (Brazil sample κ = .81, Hong Kong sample κ = .87; [261]). Articles utilizing peak-experiences indicate that universal narratives may exist. Studies investigating peak-experiences across samples in Hong Kong, India, Japan, and Norway all reported that peak-experiences relating to interpersonal joy (family, friends, romantic partners, etc.) are most frequently reported [247,262–264]. This finding was replicated in a cross-cultural study, that found 70.5% of participants from Hong Kong and 51.4% of participants from Brazil reported interpersonal joy themed peak-experiences [261]. The second most frequently reported experiences involved external achievement (e.g., winning a competition) for India and both Hong Kong samples [261,263,264], experiences relating to nature for those from Japan and Norway [247,262], and developmental landmarks for a sample of Brazilian college students [261]. One study investigating peak-experiences from travel in a sample from Mainland China reported experiences with nature, not interpersonal joy, as the most common theme [265]. In this study, interpersonal joy was the second most reported theme of peak-experiences.

### Autobiographical memory test

The AMT [248], designed to measure the ability to coherently recall memories in response to cue words (e.g., happy), was utilized in three articles. The three articles reported moderate to high inter-rater reliability (κ = .88, [266]; average reported κ = .79, [208]; average reported κ = .76, [267]). The AMT demonstrated low to moderate internal consistency in adult samples from Australia (α = .76) and Mainland China (α = .69), and high internal consistency in pre-school-aged children (Australia α = .87, Mainland China α = .90; [267]). All three articles reported cultural differences in autobiographical memory. Both Australian and Swiss adult participants reported significantly higher specific memory than Chinese adult participants [208,267], and British participants provided significantly more specific memories than Iranian participants [266]. Cultural differences in the relationships between the AMT and other personality indicators and life outcomes were also reported. For example, Swiss participants with high Schwartz traditional values (assessed by the PVQ) engaged in more deliberate grief avoidance than their Chinese counterparts, as well as both Chinese and Swiss participants with low traditional values [208].

### Thinking about life experiences scale

The TALE scale is used to measure how often individuals use autobiographical memory to direct their behavior, form social bonds, and forge self-continuity [249]. The instrument was utilized in five publications and demonstrated acceptable internal consistency across cultures, reporting low to moderate internal consistency in a Taiwanese sample [268], and moderate to high in American, Chinese, Japanese, and Trinidadian samples [259,268–271]. Confirmatory factor analysis, however, did not support the three-factor model of Self-Continuity, Social-Bonding, and Directing-Behaviour [249] in the Japanese sample (CFI = .784, RMSEA = .084; [270]). The articles reported various similarities and differences in the cultural effects on autobiographical memory functions. For example, Taiwanese and American young adults reported similar frequencies of autobiographical memory functions in forming social bonds and directing behavior; however, Taiwanese participants reported higher use of memory when maintaining self-continuity [268]. A cross-cultural study comparing people in Trinidad and the United States reported that linking past behaviors to autobiographical events was predictive of well-being in both younger and older adult Trinidadians, but only in older adult Americans [271].

### Thematic analysis of interviews

Four articles used thematic analysis of interviews and open-ended questions to explore cultural influences on various aspects of narrative identity [272–275]. An investigation of narrative identity in Mongolian and Australian women who had survived domestic violence reported that Mongolian women’s narrative identities had moved further away from the experiences of violence than Australian women [273]. Both Mongolian and Australian women shared transformations in their identities through gaining autonomy, independence, empathy, and a sense of purpose through helping others [272,273]. A narrative analysis of the experiences of immigrant mothers from North Korea and Puerto Rico reported that identity memories from childhood influenced their bicultural identity formation and parenting attitudes [275]. Interviews with children from Mainland China and the United States reported that American children described themselves using personal attributes, traits, and abstract dispositions more frequently than Chinese children, who typically described themselves by context-specific characteristics, overt behaviors, and social roles [274].

### Layer 3 summary

Metrics such as measurement invariance are not applicable to this layer and the approach to Layer 3 measurement is often determined by the goals of the research, although common narrative constructs and qualitative coding systems may be utilized. Nonetheless, the articles reviewed here suggest that cultural differences and similarities exist across narrative identities, which may provide deeper understanding about a particular individual or specific group’s personality and behaviors beyond the average pattern of the broader group.

## Discussion

The current scoping review synthesizes information regarding the applicability and utility of personality models and measures across cultures. It reviews a large number of published papers (*N *= 233) that met selection criteria from an initially identified set of 1563 published articles (Fig 1). The information arising from this review was structured according to McAdams’s framework, which defines personality across three developmental layers: (1) dispositional traits; (2) characteristic adaptations; and (3) life stories/narratives. Each layer was assessed individually to assess the cross-cultural applicability of common measures.

### Summary of findings

This review suggested that a considerable majority of these scales have been shown to possess both configural and metric invariance. This indicates that the overall factor structure and the relationship between each factor and the items loading on that factor (thus their meaning within each culture) are the same across the cultural groups tested. This suggests that the structure and meaning of these instruments can be compared across the cultural groups tested, providing substantial support for establishing the construct validity of these instruments across cultural groups. Nonetheless, the most common measurement invariance shortcoming was a failure to achieve scalar invariance. In this context, one should be cautious about making mean-level comparisons of personality traits across cultures.

From Layer 1, the measures associated with the HEXACO and Big Five models are promising as cross-cultural tools. They are supported by strong empirical evidence, demonstrating good reliability and validity, and are widely used across many settings and cultural contexts. Several Big Five measures have demonstrated measurement invariance up to the scalar and partial scalar levels across numerous national groups, suggesting that they can be used to meaningfully compare personality cross-culturally. The NEO-PI-R/NEO-PI-3 and IPIP-NEO-120 both demonstrated full scalar invariance for Neuroticism (or Emotional Stability), Extraversion, and Conscientiousness. For Agreeableness and Openness, the NEO-PI-R/NEO-PI-3 demonstrated full scalar invariance for the former and configural invariance for the latter, whereas the IPIP-NEO-120 had evidence for metric invariance for both. Both Openness and Agreeableness have not always been successfully replicated outside Western populations, so use of these measures requires caution when working with Asian and African populations. The NEO-PI-R/NEO-PI-3 has been translated into at least 40 languages or dialects [276], and 24 language versions can be purchased for use [277]. The IPIP-NEO-120 has certain advantages over the NEO-PI-R/NEO-PI-3 as it is freely available in 29 different languages.

The HEXACO model, and its related measures, should also be viewed favorably, particularly due to the inclusion of the additional personality dimension, honesty-humility. The HEXACO demonstrates some incremental validity beyond the original five traits of the Big Five model [167,175]. The primary HEXACO measure has demonstrated configural and metric invariance across 33 countries and 16 languages. Therefore, the HEXACO-PI-R can be effectively used to meaningfully study personality across several national contexts. Nevertheless, support for scalar invariance is lacking, which raises caution against making mean-level trait comparisons across countries.

The evidence for metric, but not scalar, measurement invariance (e.g., HEXACO, BFI-44) suggests that the trait concepts exist across a range of cultures, but there may be important differences in the way people respond to or their willingness to endorse items used to measure the traits. It is possible that cultural norms influence the way in which people conceptualize dispositional traits, how they describe themselves and others, and how they respond to the items presented in personality measures. Indeed, a study found that using anchoring vignettes that provided hypothetical descriptions for each personality dimension increased the structural validity of the BFI-44 in Rwandan and Filipino samples [278]. The inclusion of the vignettes was thought to provide an external benchmark of each trait that allowed for adjusting for cultural differences in responses to scale items. This suggests that use of methods like the anchoring vignette methodology could improve the cross-cultural transferability of personality instruments and comparability of personality scores. Of the emic-etic measures, the SAPI demonstrated the strongest validity and reliability and less focus on culture-specific traditions than the CPAI. The social-relational dimensions demonstrated incremental validity beyond universal traits for predicting prosocial tendencies and family orientation [124,191], indicating that it may relate more strongly to social outcomes. Nevertheless, cross-cultural studies using the SAPI are limited and measurement invariance has been established only for ethnic groups within South Africa and New Zealand. Both the SAPI and CPAI include dimensions measuring interpersonal relatedness, a trait that is likely to have relevance to cultures that strongly emphasize social obligation, interactions, and normative behavior. The CPAI may be particularly useful in assessing personality in East Asian countries, as the inclusion of traditional Confucian values in the scale may tap into core cultural aspects not included in the Big Five and HEXACO measures. The CPAI has also been found to illuminate personality characteristics of immigrants in Western countries who have retained the more traditional values and customs from their country of origin [185].

From Layer 2, the PVQ for Schwartz’s values (especially the 21-item version) hold promise from a cross-cultural perspective. This instrument has shown strong psychometric properties, including partial scalar invariance for the higher-order values of self-transcendence, self-enhancement, and conservation, and metric invariance for Openness to Change. Therefore, cross-cultural comparisons are valid for all values except Openness to Change. On the other hand, the dominant tool for measuring moral values (MFQ) is not supported at the scalar invariance level and, therefore, the comparison of mean-level scores across cultures using the MFQ should not be undertaken until stable scalar invariant models can be developed. Nonetheless, the measure has demonstrated satisfactory psychometric properties within specific cultures, including Japan, New Zealand, Russia, and the UAE. Although filial piety has rarely been used outside of Chinese-speaking countries, scales such as the FBS may provide important information regarding social aspects of personality in relevant cultures and have demonstrated partial scalar invariance across several cultural groups (Italy, Malaysia, and the U.S.).

Conversely, measures of social axioms (i.e., the SAS) have limited support for use in cross-cultural research. Partial metric invariance was supported across 23 countries (in their native languages), including the Philippines, Singapore, Peru, China, the U.S., Japan, and Ethiopia. As such, this measure may be used to look at country data individually, but the lack of scalar invariance does not support using the measure for cross-cultural comparisons. Similarly, the principal measure of “isms” (i.e., the SDI) lacks evidence of measurement invariance. Additionally, its applicability to non-Western cultures is questionable and underscored by research identifying culture-specific “isms” from China and Taiwan. As such, consideration of the population and culture of interest is required when using this measure to ensure that potentially important, culturally relevant beliefs are not missed.

Studies under the Layer 3 rubric are relatively rare and (as noted above) principally rely on qualitative analyses of autobiographical reports. As such, these measures have not been evaluated using conventional psychometric indices, including those gauging measurement invariance. Given the idiographic nature of this layer, research looking to investigate aspects of Layer 3 personality will likely need to adapt the methods of data collection and coding to reflect the research aims.

### Limitations and recommendations for future research

The search strategy used in this review excluded articles that were not available in English. This would have restricted the capacity to find non-Western measures and research. Eleven articles were excluded due to their not being available in English, and more still were likely not identified because the search terms used were in English only. Future reviews could harness the potential of AI tools and human translators to conduct a more in-depth investigation of relevant research published in languages other than English. Unfortunately, we had limited resources to employ human translators, and although AI tools are becoming more useful for translation purposes, the translation of more complex texts, such as scientific articles, still requires the intervention of human translators to ensure the quality and accuracy of the translation [279–281]. Furthermore, the ability of AI to conduct a scoping review across languages is still not well-documented.

The nature of this review focused specifically on cross-cultural comparisons of the instruments. As such, adaptations and validations of the instruments in languages different from the original instrument were not included. There are many examples of the instruments reviewed in this scoping review being translated or adapted into different languages, for example the French (e.g., [282–284]) and Danish (e.g., [285,286]) versions of BFI instruments and the Swedish version of the HEXACO-PI (e.g., [287]). Translations and adaptations of measures of characteristic adaptations, such as the French-Canadian version of the Twenty-Item Value Inventory [288]; derived from the PVQ, [289]), have also been published. Future research could aim to collate and assess international translations and adaptations of these instruments and run invariance analyses to investigate if different layers of personality (i.e., traits, characteristic adaptations, and life narratives) are measured consistently across languages. Such an investigation could determine if meaningful comparisons across scores from different translations and adaptations of the instruments can be made.

Very few articles compared the incremental validity of the instruments across cultures. Tests of incremental validity often involved comparing the Big Five dimensions to the HEXACO honesty-humility trait [175], or the additional relational traits of the SAPI [124,191] and CPAI-2 [186,290,291]. Although some evidence for incremental validity was found, further efforts should be made to compare the dispositional trait instruments to determine if the alternatives to the Big Five provide more predictive validity, particularly over shorter, more accessible instruments. Similarly, only one article compared the incremental validity of different instruments measuring characteristic adaptations [235], finding that the SAS provided a small increase in explanatory power over the SVS. When comparing instruments from different layers, two articles reported that instruments measuring characteristic adaptations provided small increases in predictive power of belief in COVID-19 conspiracy theories (MFQ compared to TIPI; [220]) and life satisfaction (SAS compared to CPAI-2; [240]) over instruments measuring dispositional traits.

The current review underscores the importance of further research to bolster the utility and validity of several personality models and instruments across cultural groups. Although most instruments have been evaluated regarding common indices of reliability and validity, the testing of measurement invariance across cultural groups is variable or lacking for many. Further invariance testing of the CPAI, MFQ, measures of filial piety, and the SDI is warranted, both because many of these instruments’ developments are culturally specific, and several of these constructs are likely to have great relevance to socio-political differences across cultures and international regions (e.g., measures of isms). Ant colony optimization has shown promise in developing cross-culturally invariant personality instruments [137,292], as well as age-invariant instruments, such as the HEX-ACO-18 [293]. As such, future research may look to ant colony optimization to identify items with cross-cultural measurement invariance.

The cross-cultural differences emerging within life stories/narratives is a novel and understudied domain. This area holds promise for further elucidating cross-cultural and linguistic differences, especially considering the extant research showing the incremental validity of this kind of assessment over and above personality traits. An interesting question arising is whether life stories/narratives could be measured using techniques amenable to psychometric analyses, which would allow for more rigorous cross-cultural comparisons of their measurement properties. Finally, a particularly important research area is the investigation of how overt behavior (including online behavior) signals underlying personality architecture, which may, in turn, robustly predict or moderate psychological processes that put people at risk for malevolent or self-injurious action. This research will be, undoubtedly, augmented by recent advances in artificial intelligence, including the use of large language models (e.g., [294]) and machine learning (for a review of how machine learning may be used in personality assessment, see [295]).

## Conclusions

This scoping review found some evidence supporting the cross-cultural validity and measurement invariance of instruments used to assess different layers of personality. In particular, the dispositional trait measures, NEO-PI-R and IPIP-NEO-120, and characteristic adaptation measure, PVQ, demonstrated the strongest evidence for cross-cultural invariance. To ensure meaningful cross-cultural comparisons of personality can be made, further testing of measurement invariance is needed to identify models and instruments that are appropriate for use within and across different cultural groups.

## Supporting information

S1 Table Glossary. (DOCX)

S1 File Raw data spreadsheet. (XLSX)
