# Supplementary material for: Understanding and assessing personality across cultures: A scoping review
Source: PLoS One. 2026 Jan 2;21(1):e0338521. doi: 10.1371/journal.pone.0338521 (PMC12758732; doi:10.1371/journal.pone.0338521)
Supplement: S1 Table — (DOCX) [file pone.0338521.s001.docx]

# Supplementary Materials 1

**S1 Table. Glossary**

| **Ant Colony Optimization** | A probabilistic optimization technique based upon the pheromone behaviour of ant colonies [1]. This technique can identify items from a long-form scale to maximize the performance (e.g., factor structure and reliability) of a new short-form scale [2]. The ant colony optimization algorithm randomly selects multiple short scales and compares them on criteria selected by the research team (e.g., measurement invariance). The items in the best performing short scale are given higher values (known as pheromone values), which increase their likelihood of being selected in the following iterations. The process of short-scale selection and comparison is repeated until the optimal shortened scale has been identified. |
| --- | --- |
| **Construct Validity** | Validity refers to the extent to which evidence supports the conclusions that can be drawn when measuring a construct. Validity is not an inherent aspect of the measure itself; rather it refers to the measure’s capacity to make a specific claim or prediction.  Construct validity refers to the extent to which an instrument accurately measures the construct it is intending to measure [3]. Theoretically, in cases of perfect construct validity (which is inevitably never achieved in practice) no discrepancy exists between the measured construct and its operational definition, when forms of bias (e.g., response bias) and experimenter effects (e.g., errors in analysis) have been controlled. |
| **Content Validity** | An instrument’s content validity is supported when items are representative of all relevant parts of the construct being measured. |
| **Convergent Validity** | Refers to evidence that an instrument’s responses have strong relationships with responses on conceptually similar measures. This can be assessed by: (1) comparing the item responses to those of an existing measure for the same construct (e.g., comparing extraversion item scores of a five-factor trait model with extraversion item scores of a six-factor trait model); and (2) comparing the item responses to those of an existing measure for a highly similar construct (e.g., examining correlations between the tradition-oriented religiousness “ism” and measures of religiosity and right-wing authoritarianism). |
| **Criterion Validity** | An instrument’s criterion validity is supported when the instrument appropriately correlates with established, comparable external variables. This category of validity is typically divided into three types: (1) *predictive validity* (evidence that the instrument correlates with a meaningful variable that is assessed after it has been administered); (2) *concurrent validity* (evidence that the instrument correlates with a meaningful variable that is assessed at a similar time to when the instrument is administered); and (3) *retrospective validity* (evidence that the instrument correlates with previously assessed variables, Horstmann et al., 2018). For example, a measure of student academic success would be considered to have criterion validity if it could predict that a student: a) will do well academically in the future; b) is currently doing well academically; and c) has done well academically in the past. |
| **‍Discriminant Validity** | Refers to evidence that an instrument’s responses diverge from (i.e., do not relate to) responses on instruments that measure distinct constructs. Discriminant validity demonstrates that the instrument accurately captures the intended construct instead of a similar but distinct construct, thereby demonstrating its specificity. |
| **Factor Analysis** | A set of mathematical procedures designed to identify latent factors within a set of data. A latent factor is the hypothetical and unobserved cause of the similar response patterns (i.e., correlations) between observed variables (e.g., scale items). By analyzing the correlation structure between observed variables, a smaller number of latent variables can be identified that explain a substantial amount of the observed data. Factor loadings represent the relationship between observed variables and the latent factors (i.e., the extent to which the variable is related to the factor). In the case of personality trait research, the observed variables are typically the personality trait items (e.g., “I make friends easily”) that correlate with other similar items (e.g., “I am the life of the party”) to reveal the latent trait (e.g., extraversion). |
| **Incremental Validity** | The improvement in prediction of a criterion obtained by adding the instrument to another source of data [5]. This means that one measure (e.g., a new measure of a personality construct) improves upon another measure (e.g., an existing measure of the same personality construct) in its ability to predict additional variance in relevant criteria over and above that measure. |
| **Measurement Invariance** | Measurement invariance is a set of factor analytic techniques used to demonstrate whether a measure is psychometrically equivalent across groups or time and is considered strong evidence as to whether an instrument measures similar constructs (e.g., a personality trait or value) across groups of interest for the use of instruments across groups of people (e.g., high extraversion has the same meaning in group A as it does in group B) [6]. If measurement invariance is supported, group means and correlations with external variables can be meaningfully compared across groups. If measurement invariance is not supported, then comparisons between groups are biased, and the measure cannot be legitimately applied across cultures. There are four main types of measurement invariance: configural, metric, scalar, and residual (explained in more detail below). |
| 1. **Configural Invariance** | Configural invariance demonstrates whether the construct has the equivalent factor structure and pattern of item loadings across groups [7]. It is the first step in invariance testing. Achieving configural invariance indicates that the construct being measured has the same factor structure across groups. |
| 1. **Metric Invariance** | Metric invariance demonstrates whether the magnitudes of item loadings on factors are equivalent across groups [7]. This is the second step in invariance testing. Achieving metric invariance indicates that the latent factors have the same meaning and are consistent across groups. |
| 1. **Scalar Invariance** | Scalar invariance demonstrates whether the mean differences in the latent factor between groups reflects true differences in the constructs being measured [7]. This is the third step in invariance testing and involves constraining the item intercepts to be equivalent between groups to ensure measurement is on the same scale. If scalar invariance is obtained, it indicates that any obtained differences between groups are real. |
| 1. **Residual Invariance** | Residual invariance demonstrates whether the sum of the item variance not shared with the latent factor (specific variance) and the measurement error (error variance) is similar across the groups [7]. This is the fourth, and often final, step when conducting invariance testing. This step is not considered essential and is not frequently implemented because it relates to aspects of measurement that are not crucial for group comparisons. |
| **Partial Invariance** | Partial invariance can occur at any stage above configural and refers to a situation when only a subset of items is invariant at that level. |
| **Principal Components Analysis (PCA)** | A variable reduction method that reduces the number of observed variables (e.g., scale items) to a smaller number of principal components (combinations of the initial variables) that can account for a considerable amount of the observed variables’ variance. This approach differs from factor analysis in that it does not try to describe the underlying model that gave rise to the observations in the data. Instead, PCA aims to reduce the complexity of the data without generalizing to a broader theoretical model. |
| **Reliability** | Reliability refers to the degree to which the scores of a measure are consistent across time periods (*test-retest reliability*), observers or raters (*inter-rater reliability*), or items (*internal consistency*). Well-known indices of internal consistency are Cronbach’s alpha and McDonald’s omega. Typically, internal consistency values of over .70 are deemed satisfactory. Nonetheless, values of less than .70 can also be acceptable for shorter scales. |

## References

[1] Deneubourg JL, Pasteels JM, Verhaeghe JC. Probabilistic behaviour in ants: A strategy of errors? Journal of Theoretical Biology 1983;105:259–71. doi:10.1016/S0022-5193(83)80007-1.

[2] Olaru G, Danner D. Developing cross-cultural short scales using ant colony optimization. Assessment 2021;28:199–210. doi:10.1177/1073191120918026.

[3] Cronbach LJ, Meehl PE. Construct validity in psychological tests. Psychological Bulletin 1955;52:281–302. doi:10.1037/h0040957.

[4] Horstmann KT, Knaut M, Ziegler M. Criterion Validity. In: Zeigler-Hill V, Shackelford TK, editors. Encyclopedia of Personality and Individual Differences, Cham: Springer International Publishing; 2018, p. 1–3. doi:10.1007/978-3-319-28099-8_1293-1.

[5] Hunsley J, Meyer GJ. The incremental validity of psychological testing and assessment: Conceptual, methodological, and statistical issues. Psychological Assessment 2003;15:446–55. doi:10.1037/1040-3590.15.4.446.

[6] Widaman KF, Reise SP. Exploring the measurement invariance of psychological instruments: Applications in the substance use domain. In: Bryant KJ, Windle M, West SG, editors. The science of prevention: Methodological advances from alcohol and substance abuse research., Washington: American Psychological Association; 1997, p. 281–324. doi:10.1037/10222-009.

[7] Putnick DL, Bornstein MH. Measurement invariance conventions and reporting: The state of the art and future directions for psychological research. Dev Rev 2016;41:71–90. doi:10.1016/j.dr.2016.06.004.
